# Noise reduction of diffusion tensor images by sparse representation and dictionary learning

**DOI:** 10.1186/s12938-015-0116-3

**Published:** 2016-01-13

**Authors:** Youyong Kong, Yuanjin Li, Jiasong Wu, Huazhong Shu

**Affiliations:** Lab of Image Science and Technology, School of Computer Science and Engineering, Southeast University, Nanjing, China; Key Laboratory of Computer Network and Information Integration, Southeast University, Ministry of Education, Nanjing, China; Department of Computer, Chuzhou University, Chuzhou, China

**Keywords:** Diffusion tensor imaging, Image denoising, Sparse representation, Dictionary learning

## Abstract

**Background:**

The low quality of diffusion tensor image (DTI) could affect the accuracy of oncology diagnosis.

**Methods:**

We present a novel sparse representation based denoising method for three dimensional DTI by learning adaptive dictionary with the context redundancy between neighbor slices. In this study, the context redundancy among the adjacent slices of the diffusion weighted imaging volumes is utilized to train sparsifying dictionaries. Therefore, higher redundancy could be achieved for better description of image with lower computation complexity. The optimization problem is solved efficiently using an iterative block-coordinate relaxation method.

**Results:**

The effectiveness of our proposed method has been assessed on both simulated and real experimental DTI datasets. Qualitative and quantitative evaluations demonstrate the performance of the proposed method on the simulated data. The experiments on real datasets with different b-values also show the effectiveness of the proposed method for noise reduction of DTI.

**Conclusions:**

The proposed approach well removes the noise in the DTI, which has high potential to be applied for clinical oncology applications.

## Background

Diffusion tensor imaging (DTI) has become a promising technique for characterizing the effects of developmental, aging and pathological processes of the central nervous system (CNS) in the tissue micro-structure and organization [1, 2]. The powerful technique has been widely applied for oncology diagnosis and treatment [3]. However, the low quality image could affect the accuracy of diagnosis and the effect of the treatment. As a result, it is essential to devise a reliable method to reduce noise for DTI.

In the past decade, a number of approaches have been developed to reduce noise for DTI. In general, these methods can be categorized into two main types, including regularization of the complex tensor fields and denoising of the scalar diffusion weighted imaging (DWI) volumes. The straight forward strategy is to perform regularization directly on the tensor fields. There is a 3 × 3 symmetric positive diffusion tensor at each voxel for DTI [4]. Several regulation methods have been designed for the complex images. Frandsen et al. [5] utilized the information of fiber orientations to develop a Bayesian method for regularizing the diffusion tensor field. However, the work proposed may be easily trapped in locally optimal solutions, which required a large of iterations to converge. Gur and Scochen [6] transferred the symmetric and positive-definite tensor into Riemannian space for regularization. To avoid the high computational complexity in Riemannian approach, an effective Log-Euclidean metric was proposed to regularize the tensor value images [7]. Regularization of complex tensor field has advantages of smaller bias and easier characterization.

In recent years, plenty of efforts have been made for reducing the Rician noise in DWI. Wirestam et al. [8] proposed a wiener like filtering method for high b-value DWI denoising. Wiest-Daessléet al. [9] developed an efficient denoising method for DWI based on non-local means variants. Tristán-Vega et al. [10] proposed an effective denoising approach by incorporating the joint information among DWI at different directions. Lam et al. [11] advanced a novel algorithm based on low rank and edge constraints to remove noise of DWI volumes. The promising theory of sparse representation was introduced by Bao et al. [12] to denoise cardiac DTI, which effectively removed the noise with preserving the contrast. The performance of sparse representation applications has been demonstrated to be highly related to the dictionary. The predefined dictionary in the approach proposed by Bao et al. may not well capture the intrinsic features of images, which thus affect the denoising performance.

In this paper, we present a novel sparse representation based denoising method for 3D DTI by learning adaptive dictionary with the context redundancy between neighbor slices. In order to capture intrinsic features of DWI images, dictionary learning is introduced to learn adaptive dictionaries from the noisy images. With the context redundancy among adjacent slices at the DWI volumes, higher redundancy could be achieved to train sparsifying dictionaries for better description of image content with and lower computation complexity. With training dictionary in a number of slices with the context redundancy, an adaptive dictionary is supposed to be obtained to enable sparser representation of the selected slices. The proposed method incorporates the sparsity signal modeling and redundancy between adjacent slices for denoising 3D DTI. The performance of our proposed method is evaluated on both simulated and real datasets with qualitative and quantitative comparisons.

## Methods

### Sparse representation

Sparse representation has become a powerful and promising modeling tool, which has been widely applied to the areas of machine learning, signal and image processing [13, 14]. The model suggests that a given signal could be sparsely represented over a specific redundant dictionary. It can be describe as an optimization problem,

1 $$\mathop {\hbox{min} }\limits_{\alpha } \left\| \alpha \right\|_{0} \, subject \, to \, \left\| {\Psi \alpha - x} \right\|_{2}^{2} \le \upepsilon$$

where $x \in \Re^{n}$ represents the signal, $\Psi \in \Re^{n \times k} (k > n)$ stands for the overcomplete dictionary, $\upepsilon$ is the bounded representation error and $\alpha$ is the representation coefficients. The notation $\left\| \alpha \right\|_{0}$ denotes the non-zero entries in the coefficients. The sparsity modeling has been demonstrated in multiple magnetic resonance imaging applications, including image reconstruction, segmentation and disease classification [15, 16].

### DWI sequence denoising using sparse representation

In diffusion imaging, a sequence of DWI volumes is acquired to quantify the water diffusion information at each voxel. The sparse representation based denoising method developed for DWI performed the processing on each 2D image independently with a predefined dictionary. The denoising model can be formulated as

2 $$\mathop {\hbox{min} }\limits_{\alpha } \left\| \alpha \right\|_{0} \, subject{\kern 1pt} {\kern 1pt} to{\kern 1pt} {\kern 1pt} \left\| {y - \Psi \alpha } \right\|_{2}^{2} \le Cn^{2} \sigma^{2}$$

where $y$ is the noisy image, $C$ is constant value and $\sigma$ is the standard deviation of Rician noise. The optimization problem could be achieved by solving an unconstrained problem

3 $${ \arg }\mathop {\hbox{min} }\limits_{\alpha } \left( {\left\| {{\text{y}} - {{\Psi }}\alpha } \right\|_{2}^{2} + \mu \left\| \alpha \right\|_{0} } \right)$$

where $\mu$ is the penalty factor.

The performance of sparse representation applications strongly depends on the sparsity level of the signal in the dictionary. Compared to predefined dictionaries from classical transforms, learned dictionary could enable maximally sparse representation of the input training signal, which has been demonstrated in several magnetic resonance imaging applications [17]. Several dictionary learning approaches [18, 19] have been developed to obtain adaptive dictionaries for numerous applications of signal processing and computer vision. Among these methods, the effective K-SVD learning method proposed by Elad et al. [18] has been demonstrated to be effective and efficiency in plenty of applications. In this study, this K-SVD method will be employed to learn adaptive dictionary direct from the noisy DWI images.

In the K-SVD learning approach, dictionary is learned from image patches of the original noisy image. The latent clean image then could be restored from the learnt dictionary. The above optimization problem will be changes to be

4 $${ \arg }\mathop {\hbox{min} }\limits_{\alpha ,x} \left( {\lambda \left\| {y - x} \right\|_{2}^{2} + \sum\limits_{i,j} {\left\| {{\text{R}}_{ij} x - {{\Psi }}\alpha_{ij} } \right\|_{2}^{2} } + \sum\limits_{i,j} {\mu_{ij} \left\| {\alpha_{ij} } \right\|_{0} } } \right)$$

where $x$ is the latent clean DWI images, $R_{ij}$ is a matrix to extract the image patches at location $[i,j]$, $\alpha_{ij}$ is the corresponding representation coefficient, $\lambda$ and $\mu$ are the penalty factors. The first term is the proximity between noisy and clean images. The second terms denote the sparse representation approximation of image patches and the last terms is sparsity requirement of the representation coefficient.

**Fig. 1:**
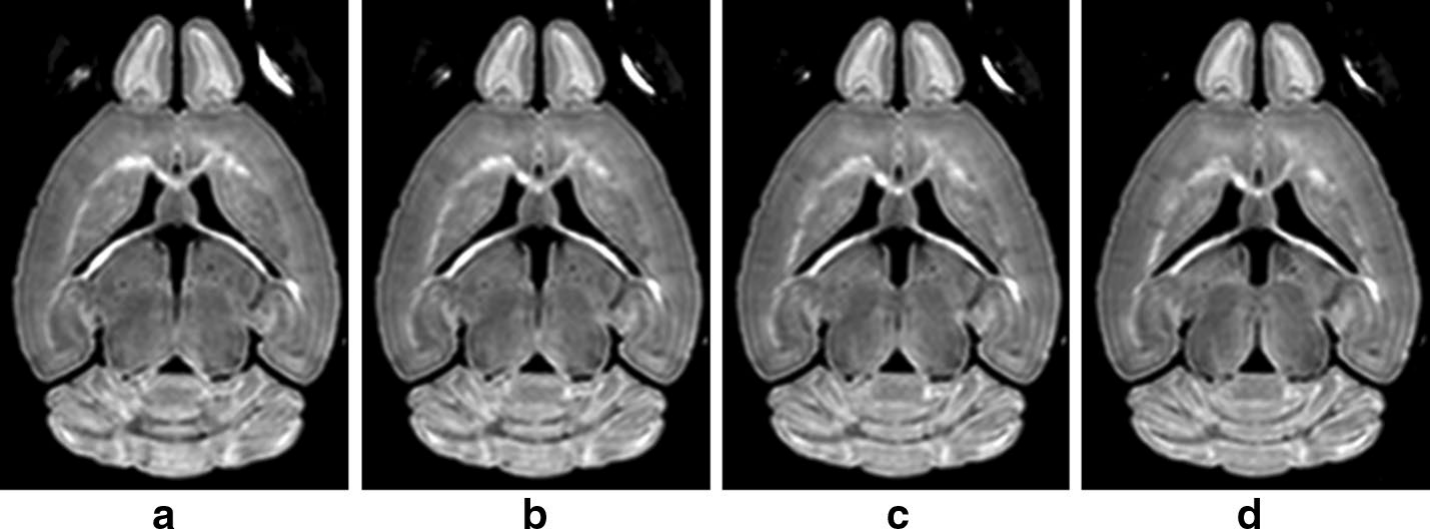
Consecutive slices of a diffusion weighted image volume. **a**–**d** are consecutive slices derived from a three dimensional diffusion weighted image

The 3D DWI volumes have similar contents and structures between adjacent slices, which can be obviously seen from Fig. 1. The corresponding learnt dictionaries for the consecutive slices are expected to be similar. Such context redundancy could be took advantage for providing more samples for training dictionary. The corrupted structure in one slice may be restored using the information from adjacent slices. Therefore, instead of training dictionaries for each slice independently, one dictionary will be learnt for a number of slices to denoise these slices simultaneously. Eq. (4) can be rewritten as

5 $${ \arg }\mathop {\hbox{min} }\limits_{\alpha ,X} \left( {\lambda \left\| {Y - X} \right\|_{2}^{2} + \sum\limits_{i,j,l} {\left\| {{\text{R}}_{ijl} X - {{\Psi }}\alpha_{ijl} } \right\|_{2}^{2} } + \sum\limits_{i,j,l} {\mu_{ijl} \left\| {\alpha_{ijl} } \right\|_{0} } } \right)$$

where $Y = \left[ {Y_{1} ,Y_{2} , \cdots ,Y_{L} } \right]$ and $L$ is the number of selected adjacent images for denoising. Several greedy methods have been proposed to solve the NP-hard $\ell_{0}$ norm problem for achieving the approximation solutions. The $\ell_{0}$ norm can also be transferred to convex relaxation $\ell_{1}$ optimization, which can be efficiently solved [20]. The convex optimization has been demonstrated to produce better quality with learned dictionary. Thereby, the above optimization problem is converted to the convex problem as

6 $${ \arg }\mathop {\hbox{min} }\limits_{\alpha ,X} \left( {\lambda \left\| {Y - X} \right\|_{2}^{2} + \sum\limits_{i,j,l} {\left\| {{\text{R}}_{ijl} X - {{\Psi }}\alpha_{ijl} } \right\|_{2}^{2} } + \sum\limits_{i,j,l} {\mu_{ijl} \left\| {\alpha_{ijl} } \right\|_{1} } } \right)$$

By training dictionary from sufficient samples, a better dictionary is supposed to be obtained to capture the intrinsic underlying features of the selected slices. All the selected slices will be denoised simultaneously with the learnt dictionary. The dictionary learned from the current image sequence will be utilized as the initial dictionary for images of next image sequence. This will highly reduce the iterations of the dictionary training process and thus highly reduce the computing time compared to learning dictionary on each 2D image independently.

### Numerical solution for the denoising problem

The complex optimization problem in equation [5] is solved using an iterative block-coordinate relaxation method. The dictionary ${{\Psi }}$ and latent clean image sequence X will be optimized through a number of training iterations. At each iteration, it consists of a sparse coding stage which obtains the sparse coefficients and a dictionary training stage that updates the atoms.

In the sparse coding process, the dictionary and latent clean image sequence $X$ are fixed. An initial dictionary $\Psi$ is generated from a specific transform and the clean image is given by the noisy DWI image sequence. The discrete cosine transform is utilized as the initial dictionary in this paper. A number of sparse coding problems will be solved using the form

7 $$\mathop {\hbox{min} }\limits_{\alpha } \left\| \alpha \right\|_{1} \, subject{\kern 1pt} {\kern 1pt} to{\kern 1pt} {\kern 1pt} \left\| {R_{ijl} - \Psi \alpha } \right\|_{2}^{2} \le Cn^{2} \sigma^{2}$$

at image patches for each location $[i,j]$ at the *l*-th slice. The efficient Lasso (least absolute shrinkage and selection operator) method is adopted to obtain the sparse representation of image patches over the dictionary [21].

During the dictionary training stage, each atom is improved sequentially with the K-SVD algorithm. For the *m*-th atom, we first identify the set of patches that use such atom. The representation error $E_{m}$ is then computed for the selected patches by removing the *m*-th atom. Singular value decomposition (SVD) is performed on the error matrix by $E_{m} = U\Delta V$. The first column of $U$ is then chosen as the updated dictionary column. The representation coefficients are updated by the entries of $V$ at the same time.

After several iterations of calculating representation coefficients vectors and training dictionaries, these two parameters are fixed. The noise free DWI image sequence can be computed by minimizing Eq. (6), which transfers to optimization problem

8 $$\text{argmin} \left( {\lambda \left\| {Y - X} \right\|_{2}^{2} + \sum\limits_{i,j,l} {\left\| {R_{ijl} X - \Psi \alpha_{ijl} } \right\|_{2}^{2} } } \right)$$

This equation can be easily solved by weighting the represented image patches as

9 $$x_{l} = \left( {\lambda I + \sum\limits_{i,j} {R_{ijl}^{T} } R_{ijl} } \right)^{ - 1} \left( {\lambda y_{l} + \sum\limits_{i,j} {R_{ijl}^{T} \Psi \alpha_{ijl} } } \right)$$

for each slice.

## Results and discussion

### Simulated datasets

Diffusion weighted imaging datasets were simulated using an diffusion tensor atlas of an adult mouse from the Biomedical Informatics Research Network Data Repository [22]. A sequence of DWI volumes was generated based on the DTI model in each voxel. Thirty-three DWI volumes were generated, including one volume with zero b-value and thirty-two images with a b-value of 1000 s/mm^2^ at different directions. Five slices of images were acquired with the spatial resolution of 256 × 256. Independent Rician noise was then added to the above produced clean images. The standard deviation of noise was set to 1/10 of the mean intensity in the center region of the DWI with zero b-value.

**Fig. 2:**
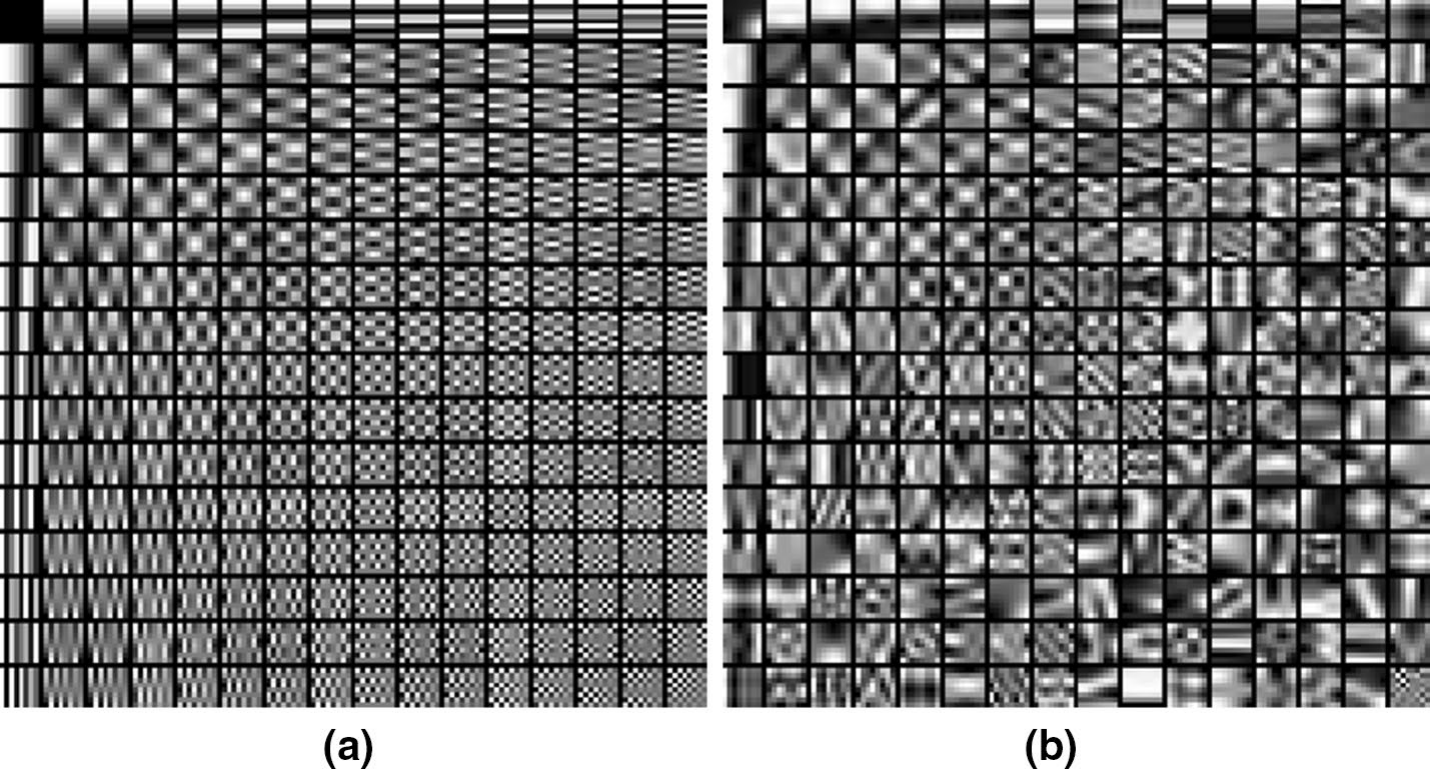
Initial and learned dictionary for simulated datasets. **a** is the initial dictinoary and **b** is the learned dictionary

In the dictionary learning process, too large image patch size can lead to a small number of training samples, and too small image patch could lead to a high computational burden. The commonly utilized image patch size is ranging from 5 × 5 to 8 × 8 [18, 23]. In this experiment, the image patch size was 8 × 8 and the dictionary size is 64 × 256. The sparsity of the representation for each patch was set to 5 and the constant C value is 1.2. Initial dictionary was given by the discrete cosine transform. Fifteen iterations were performed to learn the dictionary over the images. The effectiveness of the proposed method is compared state-of-the-art multi-component nonlocal means (MNLM) algorithm [24]. This method utilizes nonlocal means filters to images by filtering kernels on image blocks [25]. The parameters of the method have been experimentally optimized to produce the best denoising results. In addition, we also give the results of sparse representation based denoising method (SR) by learning dictionary from current slice for comparison. Figure 2 shows the initial dictionary and the learnt dictionary from the stimulated DWI image sequence by K-SVD method. Compared to initial dictionary, the learnt dictionary can capture the intrinsic features, which can better represent the DWI.

**Fig. 3:**
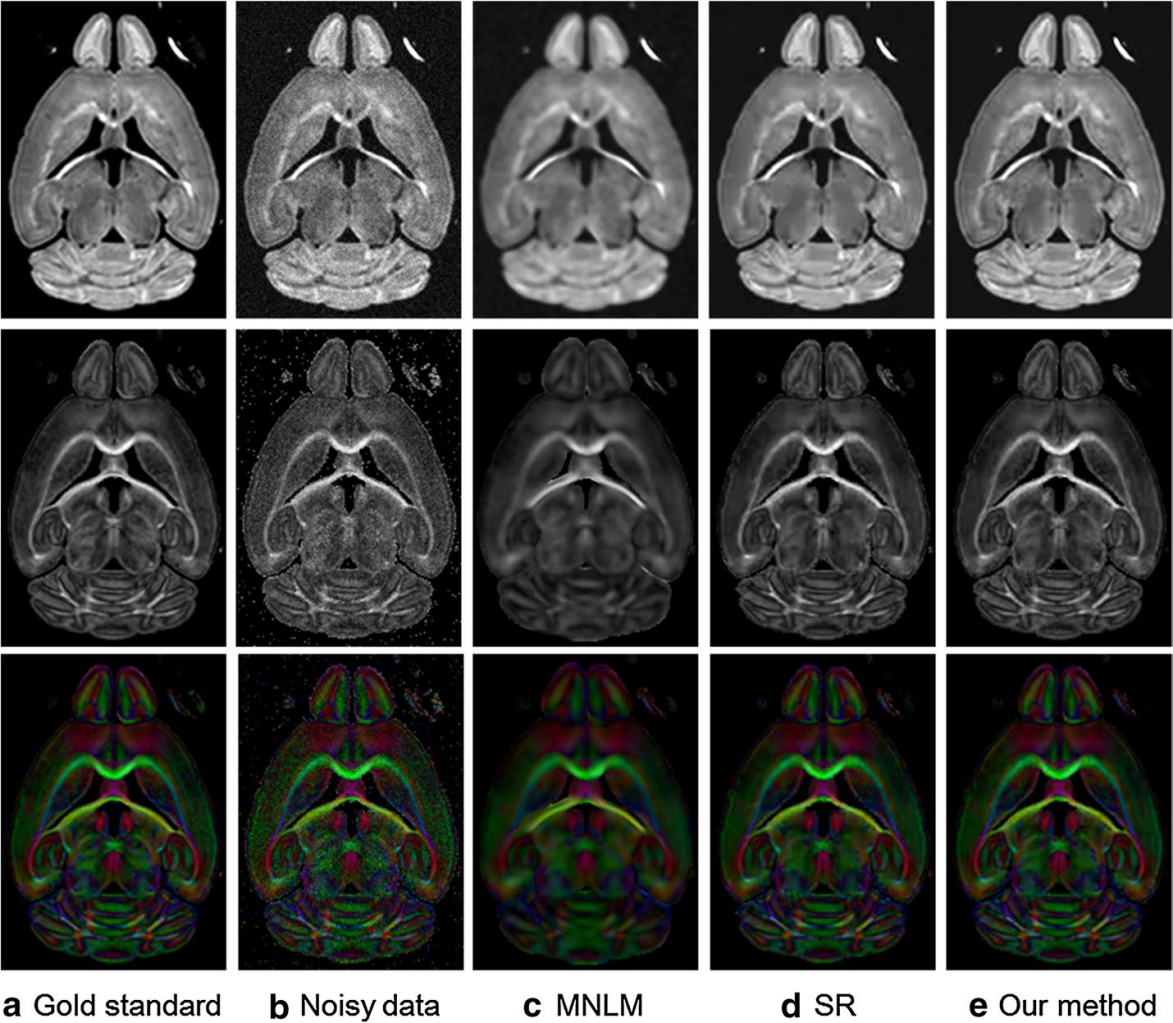
Denoising results for simulated datasets. The first row is the original diffusion weighted image. The second and third rows are the fractional anisotropy maps. The column **a** is the original gold standard and the column **b** is the noisy data. The column **c**, **d** and **e** are the denoising results using the MNLM, SR and our proposed method

The high dimensional structure of diffusion tensor makes it difficult for intuitive visualization. For easy inspection, it is appropriate to assess the effectiveness by visualizing the original DWI image and scalar maps. For DTI, fractional anisotropy (FA) maps and colored FA maps are the two important maps in clinical use and scientific research. Therefore, these three types of images are visualized for evaluation. The diffusion tensors were calculated using the least square method and the FA and colored FA maps were then computed from the DTI. Figure 3 shows one representative DWI image, corresponding fractional anisotropy (FA) maps and colored FA maps of the clean image, noisy image, the MNLM method, SR approach and our proposed method for the simulated data. The colors in the maps represent the principal diffusion direction of water at each voxel. Read, green and blue represent the directions of left–right, anterior-posterior and superior-inferior respectively. As can be seen, the denoising results from the MNLM method look good visually but with over-smoothing in several regions. Compared to MNLM, the results from SR and our proposed method obtain better results with recovering important features corrupted by noise. This demonstrates the effectiveness of the sparse representation model. Compared to SR, the results derived from our approach have better contrasts with recovering important features, which can be seen especially on the colored FA images.

**Fig. 4:**
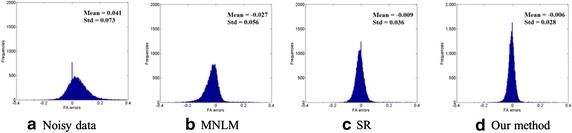
Quantitative comparison of different denoising methods. **a**–**d** are the fractional anisotropy errors of noisy data and denoising results using MNLM, SR and our proposed method

We further performed quantitative experiments to evaluate the performance of our proposed DTI noise reduction algorithm. The FA errors were computed between the clean FA maps and the results derived from the different denoising algorithms. The results of the three approaches are illustrated in Fig. 4. The MNLM obtains the worse result with largest bias (−0.027) and variance (0.056). Our approach achieves the lowest bias (−0.006) and variance (0.028).

**Fig. 5:**
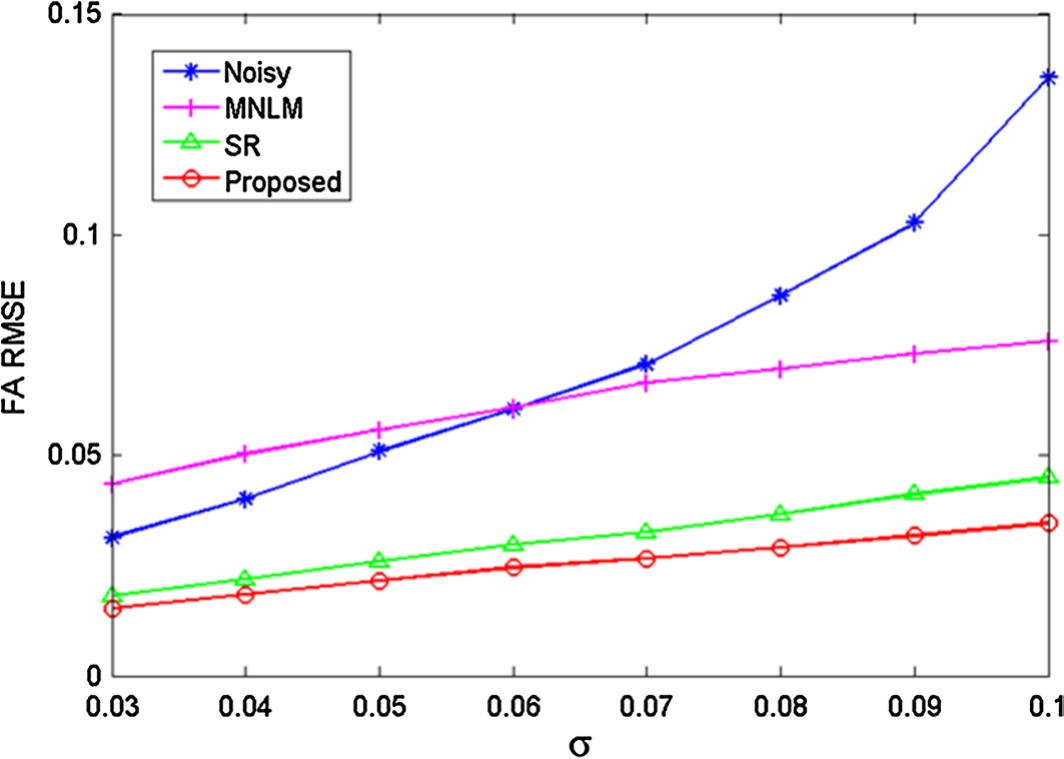
Quantitative comparison of different denoising methods with different noise levels

Moreover, the root mean squared error is calculated to evaluate the robustness of different approaches under different levels of noise. The root mean squared error is defined for the estimated FA values, which is computed as

10 $$RMSE_{FA} = \sqrt {\frac{{\sum\nolimits_{q} {(FA_{q} } - \widehat{FA}_{q} )^{2} }}{Q}}$$

where $Q$ is the total number of pixels of the non-background regions, $FA$ and $\widehat{FA}$ are the FA values estimated from the clean image and the images from different denoising methods. Figure 5 gives the quantitative comparison of FA maps between different methods under different noise levels. As can be seen, the curves of the proposed method gain more accurate diffusion parameter estimation.

Both qualitative and quantitative experiments demonstrated the superiority of our proposed algorithm compared the two classical DTI denoising methods. The MNLM method utilized the nonlocal means filters, which may produce over-smoothing results. Compared to the SR algorithm, the context redundancy between adjacent slices is utilized to train an adaptive dictionary, which can better describe the image content and intrinsic features. Therefore, the original clean images can be well obtained with higher contrasts using our proposed approach.

### Real datasets

The performance of the proposed denoising method was also further evaluated on real DTI datasets. The datasets were obtained from the diffusion imaging group at the Danish Research Centre for Magnetic Resonance, the MR Department at the Copenhagen University Hospital [26]. In vivo monkey brain DTI datasets was acquired from a 4.7 T Varian Inova MR scanner using a diffusion weighted pulse gradient spin eco sequence with single line readout. DWI datasets included 3 image with b = 0 and 90 non-collinear directions on the unit-shell with two types of b-values 1931, 3091 s/mm^2^. Three slices were obtained with the matrix size = 256 × 128, voxel size = 0.4 × 0.4 × 0.4 mm^3^, gap = 2 mm, repetition time = 5000 ms and echo time = 60 ms.

**Fig. 6:**
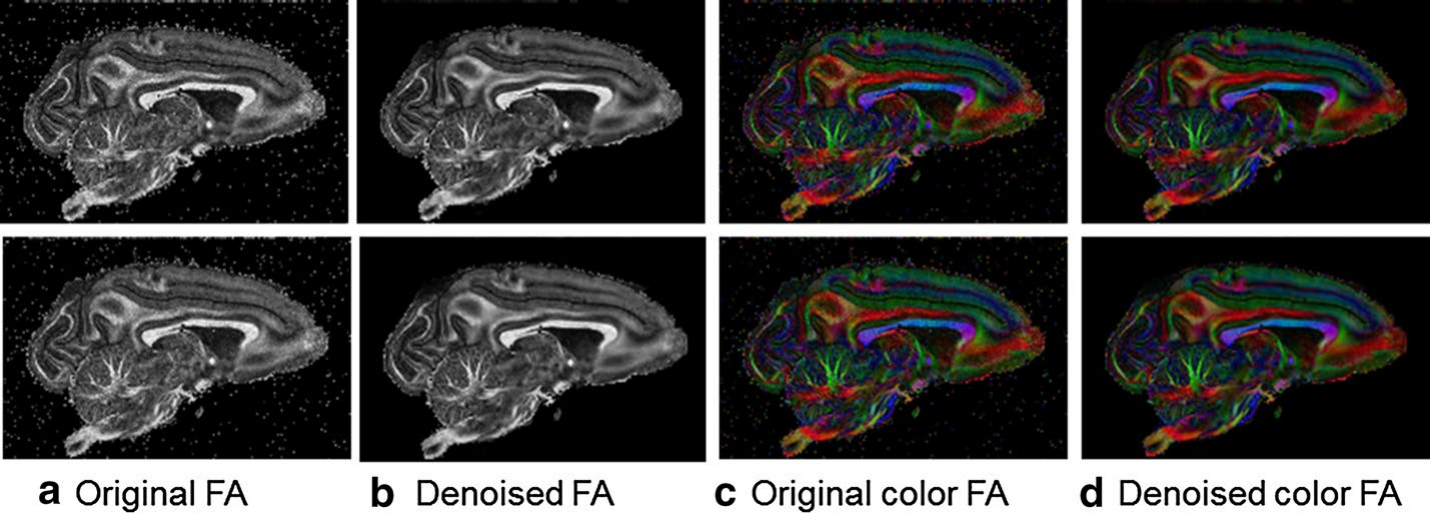
FA maps of the denoising results for real datasets. The first and second rows are the denoising results for DTI datasets with b values of 1931 and 3091 respectively. The column **a** and **b** are the original fractional anisotropy maps and the denoised maps. The column **c** and **d** are the original and denoised color fractional anisotropy maps

One b0 image and 31 images with nonzero b values were randomly selected from the 93 images to evaluate the denoising algorithm. Due to the small size of the real images, the image patch size was set to 6 × 6 to enable a sufficient number of training samples, and the dictionary size was 36 × 100. Figure 6 illustrates the denoising results of our proposed method for one slice of the vivo DTI data with two different b values. Scalar and colored FA maps were shown to evaluate the effectiveness. Gold standard cannot be available for the real datasets. However, qualitative improvement can be easily seen from these maps. Some structures were contaminated by noise before denoising, especially for higher b values. After noise reduction with our proposed method, better definition of these corrupted structures was achieved with better contrasts.

## Conclusions

We have proposed an effective denoising method for 3D DTI by combining the sparse representation and dictionary learning. The proposed approach has two desirable advantages. At first, our method leverages the powerful K-SVD algorithm to learn adaptive dictionary for maximal sparse representation of the image. Compared to specified dictionary from traditional transforms, adaptive learned dictionary could better describe the image content and intrinsic features. Second, the context redundancy existed among adjacent slices of 3D DWI volume is incorporated into the sparse representation based denoising model to achieve higher sparsity with lower computational complexity. Similar structures are always existed in the neighbor slice of the three dimensional images. Such redundancy could be utilized for providing more samples for better dictionary learning. Both the qualitative and quantitative evaluations on stimulated and real datasets demonstrate the performance of our proposed method for DTI noise reduction. The proposed approach well removes the noise in the DTI, which has high potential to be applied for clinical applications. One possible limitation of the proposed approach is the relatively high computational time compared to other classical denoising algorithm for the high dimensional DTI datasets. More time is required to optimize the dictionary in the sparse representation model. Our algorithm has high potential to be accelerated using the multiple cores and the advanced graphic processing unit. Information on patch based feature distinctness in different scales will also be considered to be incorporated to enhance the filtering performance [27, 28]. Besides, the powerful supervoxel technique has a high potential to be introduced to accelerate the denoising algorithm [29].

## Abbreviations

DTI: diffusion tensor image
DWI: diffusion weighted image
FA: fractional anisotropy
SVD: singular value decomposition
